# Identification of Targets of CUG-BP, Elav-Like Family Member 1 (CELF1) Regulation in Embryonic Heart Muscle

**DOI:** 10.1371/journal.pone.0149061

**Published:** 2016-02-11

**Authors:** Yotam Blech-Hermoni, Twishasri Dasgupta, Ryan J. Coram, Andrea N. Ladd

**Affiliations:** 1 Department of Cellular and Molecular Medicine, Lerner Research Institute, Cleveland Clinic, Cleveland, Ohio, United States of America; 2 Program in Cell Biology, Department of Molecular Biology and Microbiology, Case Western Reserve University School of Medicine, Cleveland, Ohio, United States of America; International Centre for Genetic Engineering and Biotechnology, ITALY

## Abstract

CUG-BP, Elav-like family member 1 (CELF1) is a highly conserved RNA binding protein that regulates pre-mRNA alternative splicing, polyadenylation, mRNA stability, and translation. In the heart, CELF1 is expressed in the myocardium, where its levels are tightly regulated during development. CELF1 levels peak in the heart during embryogenesis, and aberrant up-regulation of CELF1 in the adult heart has been implicated in cardiac pathogenesis in myotonic dystrophy type 1, as well as in diabetic cardiomyopathy. Either inhibition of CELF activity or over-expression of CELF1 in heart muscle causes cardiomyopathy in transgenic mice. Nonetheless, many of the cardiac targets of CELF1 regulation remain unknown. In this study, to identify cardiac targets of CELF1 we performed cross-linking immunoprecipitation (CLIP) for CELF1 from embryonic day 8 chicken hearts. We identified a previously unannotated exon in *MYH7B* as a novel target of CELF1-mediated regulation. We demonstrated that knockdown of CELF1 in primary chicken embryonic cardiomyocytes leads to increased inclusion of this exon and decreased *MYH7B* levels. We also investigated global changes in the transcriptome of primary embryonic cardiomyocytes following CELF1 knockdown in a published RNA-seq dataset. Pathway and network analyses identified strong associations between CELF1 and regulation of cell cycle and translation. Important regulatory proteins, including both RNA binding proteins and a cardiac transcription factor, were affected by loss of CELF1. Together, these data suggest that CELF1 is a key regulator of cardiomyocyte gene expression.

## Introduction

The RNA binding protein CUG-BP, Elav-like family member 1 (CELF1) regulates cell type- and developmental stage-specific alternative splicing of cardiac transcripts [1–3]. In the developing heart, CELF1 is restricted to the myocardium, where it exhibits a strong nuclear presence [4,5]. CELF1 expression peaks in the embryonic heart during cardiac morphogenesis, then drops during fetal through adult stages [1,3,4]. It has been proposed that the decline in CELF1 after birth drives fetal-to-adult transitions in alternative splicing of CELF targets during postnatal maturation of the heart [1,3,6]. Up-regulation of CELF1 in the adult heart contributes to the pathogenic reiteration of fetal splicing patterns in myotonic dystrophy type 1 (DM1) mouse models and human patients [2,7–9]. Over-expression of CELF1 in heart muscle recapitulates many of these splicing defects and induces cardiomyopathy in transgenic mice [10,11]. Up-regulation of CELF1 and changes in CELF-mediated splicing have also recently been implicated in diabetic cardiomyopathy [12].

In addition to regulating splice site choice in the nucleus, CELF1 also regulates polyadenylation status, mRNA stability, and translation of target transcripts in the cytoplasm [13,14]. Loss of CELF1 in skeletal muscle cells is associated with increased stability of transcripts harboring CELF1 binding sites in their 3’ UTRs [15–17]. Knockdown of CELF1 in human primary T cells and HeLa cells also stabilizes transcripts containing elements that bind CELF1 in their 3’ UTRs [18,19], suggesting that CELF1 plays a general role in facilitating mRNA decay in the cytoplasm. CELF1 binding in the 5’ UTR has been shown to promote translation of a number of transcripts [13], including the cell cycle inhibitor *p21* in skeletal muscle cells [20]. Although cytoplasmic targets of CELF1 have not been characterized in heart muscle cells, a fraction of CELF1 protein in embryonic cardiomyocytes is found in the cytoplasm [3,4].

To identify targets of CELF1 in the developing heart, we first performed cross-linking immunoprecipitation (CLIP) for CELF1 from embryonic day 8 chicken hearts. We identified a previously unannotated exon in *MYH7B* as a novel target of CELF1-mediated alternative splicing regulation. Inclusion of this exon would lead to the insertion of a premature termination codon that would either produce a short, nonfunctional MYH7B peptide or destabilize *MYH7B* transcripts. Knockdown of CELF1 in chicken primary embryonic cardiomyocytes leads to an increase in exon inclusion and decrease in *MYH7B* levels. Next, we analyzed a published RNA-seq dataset to identify global changes in the transcriptome of primary chicken embryonic cardiomyocytes following CELF1 knockdown. More than 8,000 transcripts were found whose levels differed between CELF1-depleted and control cells by at least 1.5-fold, and over 3,000 that differed by at least 2-fold. Pathway and network analyses identified strong associations between CELF1 and regulation of cell cycle and translation. Changes in the expression of several regulatory factors, including both RNA binding proteins and a cardiac transcription factor, were validated by real-time RT-PCR. Together, these data suggest that CELF1 is a key regulator of cardiomyocyte gene expression at multiple levels.

## Materials and Methods

### Animal use

Fertilized Hy-line W-36 White Leghorn chicken eggs were purchased from the Department of Animal Sciences at Ohio State University. Only early-gestation (embryonic day 8) chicken embryos were used in this study, which are not subject to federal regulation and do not require approval from the Cleveland Clinic Institutional Animal Care and Use Committee. Chicken embryos were euthanized by decapitation immediately upon removal from the egg, which is consistent with the recommendations of the American Veterinary Medical Association Panel on Euthanasia for euthanasia of birds.

### Cross-linking immunoprecipitation (CLIP)

CLIP was performed using embryonic day 8 (Hamburger and Hamilton stage 35) chicken hearts as previously described [21] with a few modifications. In particular, due to a high level of endogenous RNase activity in the heart, over-digestion of RNA was observed without addition of exogenous RNase. Hence, appropriately sized CLIP tags were obtained by addition of an RNase inhibitor. Immunoprecipitation was performed using the 3B1 antibody (Santa Cruz), which specifically recognizes CELF1 with little to no cross-reactivity with other family members [3]. Additional procedural detail can be found in the S1 File.

### Analysis of CLIP tags

CLIP tags were extracted from clone sequences using an in-house script (S2 File) that identified sequence tags bound by CLIP adapters in the correct orientation, removed adapter sequences, and tabulated the resulting tags. The nucleotide composition of the tags was analyzed using an in-house script (S3 File) that counted the occurrence of each nucleotide combination (1 to 6-mer) in the tag dataset; this program made use of a script posted by Mike Golvach, 2008, shared under the Creative Commons Attribution 3.0, which generated oligonucleotide motifs. To characterize CLIP tag-containing introns, intron coordinates within annotated genes were downloaded from the UCSC genome browser [22] using the Galaxy platform [23], and compared to the genomic coordinates of the tags using an in-house script (S4 File). The same approach was used to analyze CLIP data for CELF1 [24] and Nova [25] in postnatal mouse brain using the mouse mm9/NCBI37 genome and annotation (July, 2007).

### Culture and transfection of primary embryonic cardiomyocytes

Hearts were harvested from embryonic day 8 (stage 35) chicken embryos, and primary embryonic chicken cardiomyocytes were collected as previously described [26] with minor modifications. A detailed description of the isolation and culture of chicken primary embryonic cardiomyocytes can be found in Blech-Hermoni and Ladd [27]. Briefly, hearts were serially digested with 0.13% trypsin (TRL3; Worthington Biochemicals), 0.13% collagenase (CLS-2; Worthington Biochemicals), and 0.033% DNase I (D2; Worthington Biochemicals) at 37°C with agitation. Dissociated heart cells were separated by a 1.050/1.060/1.082 g/mL Percoll (Pharmacia) gradient (with cells loaded in the 1.082 g/mL layer), and purified myocytes were isolated from the interphase between the bottom two layers following centrifugation at 2000 x g. Cardiomyocytes were plated on 0.005% Fibronectin (F1141; Sigma) at a density of 2.5x10^5^ cells per 35 mm plate. Cardiomyocytes were transfected 24 hours after plating using Lipofectamine 2000 transfection reagent (Life Technologies) and a final concentration of 100 nM siRNA. siRNA duplexes against CELF1 (“si1”: 5’-GGGUGCUGUUUUGUUACAUdTdT-3’, “si2”: 5’-GAGCCGAGGUUGUGCAUUUdTdT-3’) [28], siGLO Green or siGLO Red control siRNAs (referred to in the text as “siCont”) were purchased from Dharmacon (now Thermo Scientific). Total protein and RNA samples were collected from parallel plates 72 hr after transfection.

### RNA-seq and pathway analysis

A detailed description of the generation of the RNA-seq dataset can be found in Blech-Hermoni and Ladd [27]. Briefly, primary chicken embryonic cardiomyocytes were transfected ± CELF1 siRNA (si2) and total RNA was harvested at 72 hr post-transfection as described above. RNA quality control, library preparation, and high-throughput sequencing were performed on three mock- and three si2-transfected samples using an Illumina HiSeq 2000 platform through The Ohio State University Comprehensive Cancer Center. Subsequent read mapping and differential gene expression analyses were performed by the Case Western Reserve University Genomics Core. The genome sequence and corresponding annotation of *Gallus gallus* from Ensembl release 73 were used as the reference. Reads were mapped to the reference sequence using TopHat v2.0.9, a reference transcriptome was generated with Cufflinks v2.1.1, and differential gene expression analysis was carried out using CuffDiff. Genes with P values ≤ 0.05 after corrections for multiple testing and false discovery rate were considered significant. Raw data files of the RNA-seq reads have been deposited in NCBI’s Gene Expression Omnibus [29], and are accessible through GEO Series accession number GSE67360 (http://www.ncbi.nlm.nih.gov/geo/query/acc.cgi?acc=GSE67360).

In this study, genes identified as differentially expressed in the RNA-seq dataset were analyzed using Ingenuity Pathway Analysis (IPA) software (Ingenuity Systems). A Microsoft Excel worksheet containing all of the transcripts expressed in both mock- and si2-transfected cells that were identified as having a significant difference in gene expression by CuffDiff (regardless of the fold change of the difference) was uploaded, and these transcripts were mapped into corresponding gene objects in the Ingenuity Knowledge Base (IKB) using the available gene IDs. It should be noted that IPA is only able to map gene IDs from chicken that possess specific identifiers (i.e., dbSNP, Entrez Gene, Genbank, Genpept, NCBI GI number, Unigene, or Swissprot/Uniprot), which are mapped according to the HomoloGene to the ortholog information in the IKB. In this case, 5671 IDs were successfully mapped and 4350 failed to map due to lack of supported annotation. Those genes that exhibited ≥ 1.5-fold difference in gene expression between mock- and siCELF1-treated samples were then selected; 2617 analysis-ready molecules were identified and used for subsequent pathway and network analyses.

### Western blotting

CELF1, CELF2, and MBNL1 western blots were performed as previously described [4,30]. MBNL2 was detected with the mouse monoclonal anti-MBNL2 antibody 3B4 (Santa Cruz, catalog number sc-136167). Secondary antibodies used were goat anti-mouse-HRP (Calbiochem, catalog number DC02L) and goat anti-rabbit-HRP (Calbiochem, catalog number 401393). Membranes were stripped using Restore Western Blot Stripping Buffer (Thermo Scientific), and re-probed for GAPDH as previously described [31]. Protein integrity and equivalent loading were confirmed both by GAPDH expression and Ponceau S staining for total protein.

### Semi-quantitative and real-time RT-PCR

Total RNA was extracted using Trizol (Life Technologies) according to manufacturer’s protocols. *MYH7B* alternative splicing was assessed by semi-quantitative RT-PCR with 5’ radiolabeled primers as previously described [32] using the primers shown in Table 1 and conditions optimized for amplification in the linear range with 100 ng total RNA per reaction: 64.6°C annealing temperature, 20 cycles of amplification. PCR products were resolved on 5% denaturing polyacrylamide:urea gels. All gels were dried, scanned on a Storm 820 Molecular Imager, and quantified using ImageQuant software. The identities of PCR products were confirmed by sequencing. Total *MYH7B* transcript levels were measured in primary embryonic cardiomyocytes by real-time RT-PCR as previously described [33] using TaqMan probes (Life Technologies) for chicken *MYH7B* (FAM-labeled, Gg03337745_m1) normalized against *GUSB* (VIC-labeled, Gg03358465_m1, primer limited). Detailed methods for measuring *CELF1*, *CELF2*, *MBNL1*, and *HOPX* transcript levels by qRT-PCR can be found in Blech-Hermoni and Ladd [27]. Briefly, *CELF1*, *CELF2*, *and MBNL1* transcript levels were determined by real-time RT-PCR as previously described [4,33] using TaqMan probes for chicken *CELF1* (FAM-labeled, Gg03340922_m1) *CELF2* (FAM-labeled, Gg03364304_m1), and *MBNL1* (FAM-labeled, Gg03356554_m1) normalized against *GUSB* or *GAPDH* (VIC-labeled, Gg03346982, primer limited). *MBNL2*, *HOPX*, and *SRF* transcript levels in chicken primary embryonic cardiomyocytes were determined by real-time RT-PCR as previously described [31] using SYBR Green Master Mix (Applied Biosystems) and normalized to *GAPDH* using the primers shown in Table 1. Samples from at least three independent transfections were each run in triplicate. Error bars represent standard error of the mean values for the biological replicates. Statistical comparisons of means were performed via t-tests assuming unequal variances using Microsoft Excel software. Differences were considered statistically significant when P ≤ 0.05.

**Table 1:** RT-PCR primer sequences used in this study.

Gene	Purpose^a^	Sequence^b^	Product sizes (bp)
*MYH7B*	Alt. splicing	F: GGGAGGCTGCTGAATACCT	291, 375
		R: GCTTGAGGTTGTAGAGCACG	
*MBNL2*	qRT-PCR	F: AGTCTACAAGCGGGACTTAATG	102
		R: GGGAGGACGATTAACGATGTC	
*HOPX*	qRT-PCR	F: AGGAGCAGCATCCTCTAGTCC	140
		R: TTGCAAGGTGAACAAGCATC	
*SRF*	qRT-PCR	F: CCTCAACTCCCCTGACTCAC	150
		R: GTGAAGGTCGGTTTCAGCAC	
*GAPDH*^c^	qRT-PCR	F1: GATACACAGAGGACCAGGTTG	146
		R1: ACGGTTGCTGTATCCAAACTC	
*GAPDH*^c^	qRT-PCR	F2: CAAGAGGGTAGTGAAGGCTG	146
		R2: AATGGTCATTCAGTGCAATGC	

^a^ Alt. splicing = semi-quantitative RT-PCR for alternative splicing; qRT-PCR = real time RT-PCR for transcript levels

^b^ F = forward primer sequence, R = reverse primer sequence; all primers are shown 5’ → 3’

^c^ F1/R1 and F2/R2 primer sets were used interchangeably

## Results

### CELF1 binds predominantly to UG-rich intronic sequences in the embryonic heart

**Fig 1:**
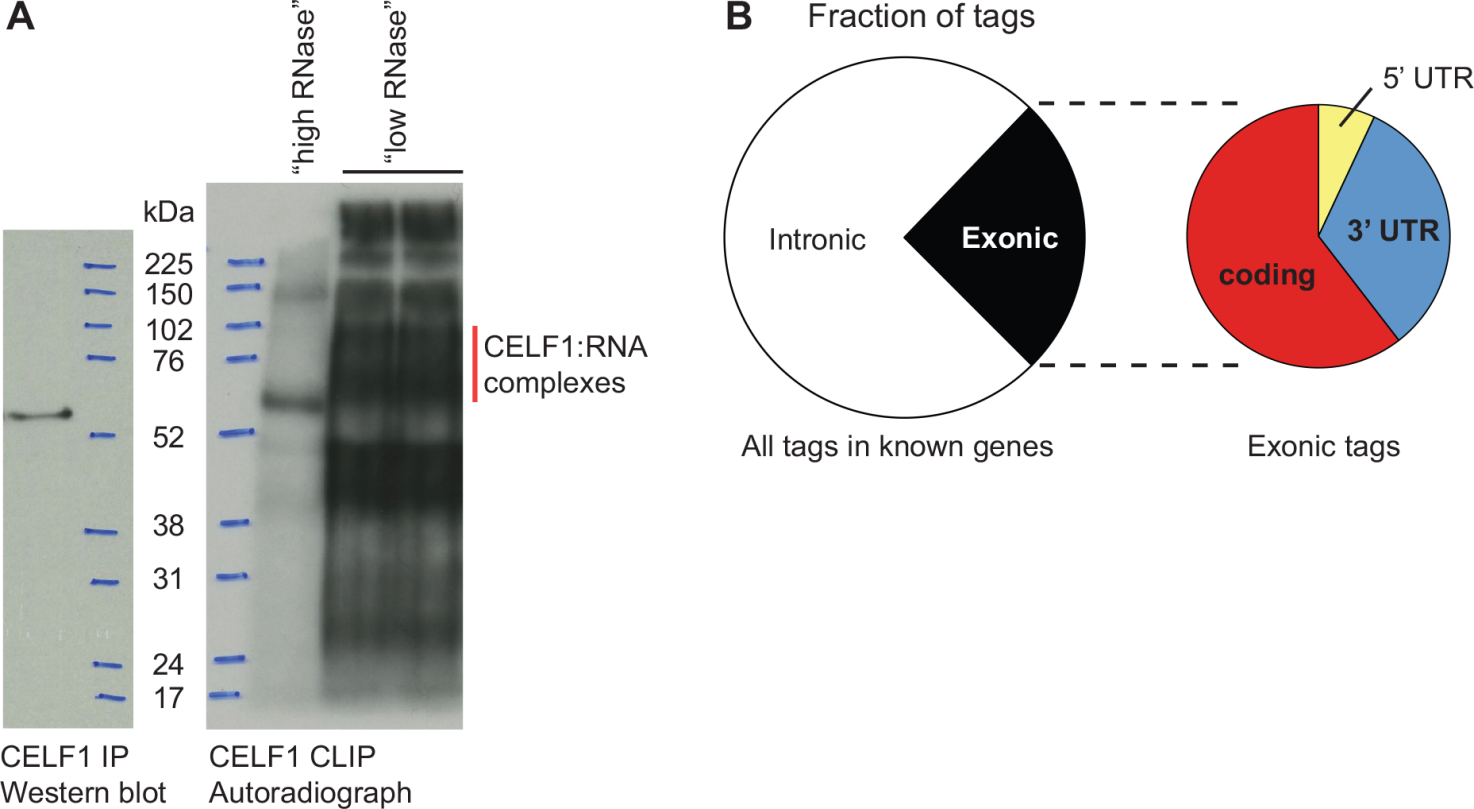
Cross-linking immunoprecipitation (CLIP) of CELF1 from embryonic chicken heart. (A) CLIP was performed on embryonic day 8 chicken hearts using an anti-CELF1 antibody. Vertical red line indicates immunoprecipitated CELF1:RNA complexes following addition of an RNase inhibitor to block high levels of endogenous RNase activity (“low RNase” lanes); fully digested complexes (“high RNase”) run just above the size of immunoprecipitated CELF1 alone (Western blot). (B) Distribution of CLIP tags within known genes.

To identify cardiac transcripts that directly bind to CELF1 *in vivo*, we performed CLIP using embryonic day 8 chicken hearts (Fig 1A). Of 564 different tags obtained, 26% (148) represented bacterial rRNA sequences (a common contaminant of CLIP), and 35% (197) could not be mapped with high confidence or mapped to more than one genomic locus. The remaining 39% (219 tags) were successfully mapped to the chicken genome using the BLAT search tool on the University of California, Santa Cruz (UCSC) genome browser [22] and/or BLAST search of the NCBI database [34]. We identified 170 tags within known genes, 13 within unknown genes (i.e., there is EST and/or cDNA evidence supporting a transcript expressed from that genomic locus, but the gene is unannotated and has no recognized homologs), and 36 within intergenic regions (S1 Table). It should be noted that due to the poor annotation of the chicken genome, the majority of genes containing tags (>70%) were not annotated in the UCSC genome browser; in these cases, homology and synteny with human and mouse orthologs were used to identify the corresponding chicken genes.

Approximately 75% of the CELF1 CLIP tags within genes mapped to introns (Fig 1B), consistent with the localization of CELF1 in the nucleus in embryonic heart muscle cells [4], and its known role as a regulator of pre-mRNA alternative splicing in the heart [1–3,7]. In other cell types, CELF1 has also been shown to regulate mRNA polyadenylation status, stability, and translation in the cytoplasm, primarily through interactions with the 5’ or 3’ UTR [16–18,20,35–38]. Surprisingly, less than half of the exonic CLIP tags were mapped to either a 5’ or 3’ UTR, but rather were found in internal coding exons (Fig 1B). Several were found within exons that are known to be alternatively spliced, raising the possibility that CELF1 regulates the alternative splicing of cardiac transcripts via both intronic and exonic binding sites.

**Fig 2:**
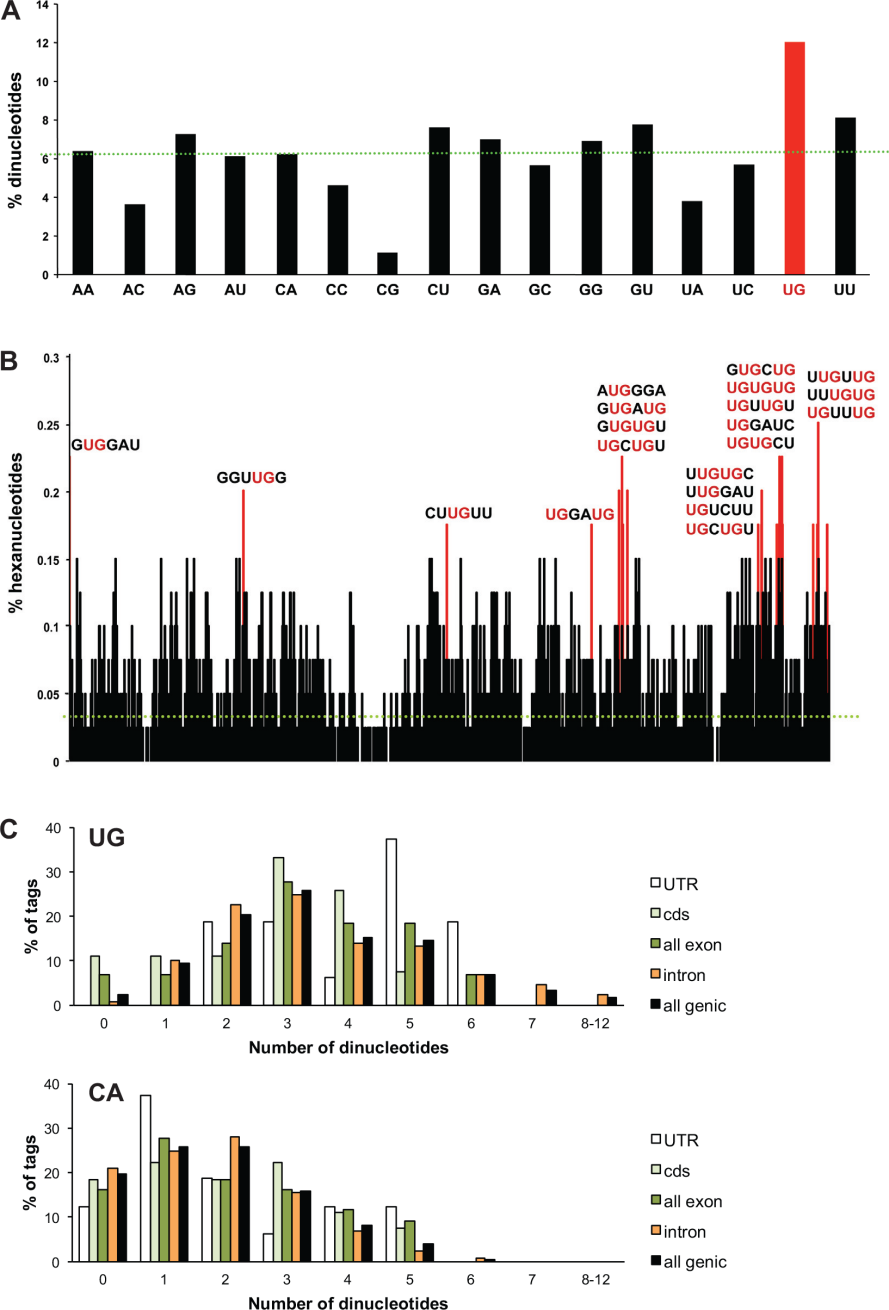
CELF1 CLIP tags are enriched with UG motifs. (A) Incidence of dinucleotides within CELF1 CLIpP tags that map to known genes. The dotted green line indicates the incidence expected if all dinucleotides were equally represented. (B) Incidence of hexanucleotides within CELF1 CLIP tags that map to known genes. The 20 most-frequent hexanucleotides are indicated in red. The dotted green line indicates the incidence expected if all hexanucleotides were equally represented. The sequences of the top hexanucleotides are shown, with UG dinucleotides within those motifs in red. (C) The distributions of tags containing different numbers of UG or CA dinucleotides are shown.

An analysis of the frequency of dinucleotides within the CELF1 CLIP tags indicates that there is an enrichment of UG dinucleotides (Fig 2A). Furthermore, all of the most frequently occurring hexamers are U- and G-rich and contain at least one UG dinucleotide (Fig 2B). This is consistent with known CELF1 binding preferences, as CELF1 has been shown to bind with high affinity to U/G-rich elements *in vitro*, in particular those containing UG dinucleotides [39–42]. Likewise, MEME analysis of CLIP tags recently obtained from mouse C2C12 myoblasts identified a consensus binding motif for CELF1 containing repeated UGU elements [15]. UG dinucleotides are not non-specifically enriched by the CLIP procedure, as UGs are not enriched in CLIP tags from an unrelated RNA binding protein, Nova, that binds to YCAY motifs ([25] and S1 Fig). The distribution of CELF1 CLIP tags containing different numbers of UG dinucleotides shows that the enrichment of UG motifs is widespread, and not the result of a subset of tags with an unusually high number of UGs (Fig 2C). In contrast, a large fraction of tags contain only one or no CA dinucleotides, which have the same G/C content, but appear with a frequency expected by random chance. The distribution of UG dinucleotides within exonic and intronic tags is similar, but amongst the exonic tags there are significantly more UG dinucleotides in tags that fall within UTRs than in those within coding regions (1-tailed *t*-test, P = 0.002).

Most well characterized splicing regulatory elements are found close to the splice site(s) they regulate. U/G-rich motifs have been found to be enriched in intronic regions proximal to cassette exons (i.e., within 200 nt) that are alternatively included in heart and skeletal muscle, and the enrichment of these putative CELF binding sites is highly conserved in sequence and position between human, mouse, chicken, and frog [43,44]. An RNA binding map recently generated for CELF1 on the basis of 24 alternative splicing events in skeletal muscle cells indicates that binding immediately upstream of an alternative exon is associated with exon skipping, whereas binding in the proximal downstream intron is associated with inclusion [15]. We found that only a small fraction of intronic CELF1 CLIP tags fall within 500 nucleotides of a known splice site. Using the subset of 46 intronic tags that fall within genes annotated in the UCSC genome browser (S2 Table), we found that less than 20% (9/46) are within 500 nt of at least one annotated splice site; only 15.2% are within 500 nt of the nearest upstream splice site, whereas 8.7% are within 500 nt of the nearest downstream splice site. Furthermore, closer examination of the introns containing these CELF1 CLIP tags revealed that most were unusually large (Table 2 and S2 Table). Although introns flanking alternative exons tend to be longer than those flanking constitutive exons, the average intron length in chick is less than 3,000 nt regardless of its position [45,46]. Only 39% of CELF1 CLIP tag-containing introns were less than 10,000 nt in length, however, and 13% were over 100,000 nt long (S2 Table). To determine whether the intron length or position of intronic binding sites in our CLIP tag set is specific to the species, developmental stage, and/or tissue type, we performed similar analyses on published CELF1 CLIP tags from postnatal mouse hindbrain [24]. As we saw in the embryonic chicken heart, a minority of intronic CELF1 CLIP tags from the postnatal mouse brain lie within 500 nt of the nearest upstream (10.1%) or downstream (6.5%) splice site, and the introns containing them are unusually large (Table 2). These observations are also not unique to CELF1. Ule and colleagues reported that almost two-thirds of intronic Nova CLIP tags from adult mouse brain were found within large (>10,000 nt) introns [25]. Analysis of the intronic Nova CLIP tags from their study shows that very few are found within 500 nt of an upstream (8.1%) or downstream (6.6%) splice site, and confirms that tag-containing introns are on average very large (Table 2).

**Table 2:** Lengths of introns containing CLIP tags and relative position of tags within introns.

Source of CLIP tags	Statistic	Intron length (nt)	Distance from upstream splice site (nt)	Distance from downstream splice site (nt)
CELF1 CLIP tags, embryonic chicken heart (this study)^a^				
	Mean	44,293 ± 8,608	21,063 ± 5,271	23,202 ± 5,600
	Median	23,569	3,718	6,804
	Range	370 to 247,578	102 to 190,843	103 to 184,011
CELF1 CLIP tags, postnatal mouse brain [24] ^b^				
	Mean	77,984 ± 11,456	32,441 ± 4,669	45,462 ± 8,298
	Median	17,712	6,342	7,244
	Range	293 to 729,767	35 to 221,376	15 to 670,693
Nova CLIP tags, adult mouse brain [25] ^b^				
	Mean	87,458 ± 9,169	33,905 ± 4,081	53,481 ± 6,976
	Median	32,058	8,309	12,524
	Range	106 to 729,767	11 to 377,919	8 to 727,004

^a^ Only intronic tags lying within chicken genes that are annotated in the UCSC genome browser were included in this analysis

^b^ All CLIP tags reported by the authors to be intronic were included in this analysis

### Alternative splicing of *MYH7B* inversely correlates with total transcript levels and CELF1 protein

**Fig 3:**
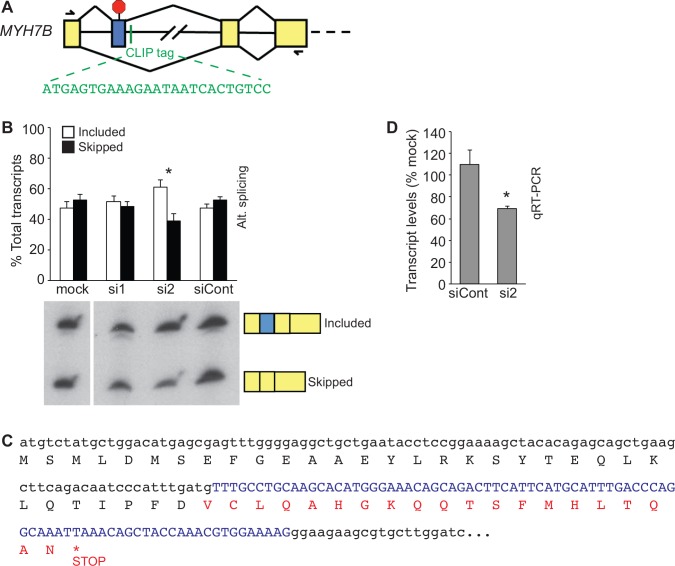
CELF1 regulates the inclusion of an unannotated exon in chicken *MYH7B* transcripts. (A) A CELF1 CLIP tag (green) maps to an intron within the coding region of *MYH7B*. RT-PCR using primers in upstream and downstream exons (indicated by half arrows) revealed the presence of a previously unrecognized exon (blue box) that is alternatively included in the embryonic heart. (B) The extent of inclusion of the novel *MYH7B* alternative exon was determined by semi-quantitative RT-PCR in primary embryonic cardiomyocytes transfected with or without control (siCont) or anti-CELF1 (si1 and si2) siRNAs. Data represent mean values from three independent transfections. A representative autoradiogram from one of the transfection sets is shown; an empty lane between the mock and si1 sample has been excised. (C) Translation of the transcript sequence including this exon indicates that its inclusion would lead to the insertion of an in-frame stop codon close to the N-terminal end of the protein. (D) Total *MYH7B* transcript levels in primary cardiomyocytes transfected with control (siCont) or anti-CELF1 (si2) siRNA were compared to mock-transfected controls by qRT-PCR. An asterisk indicates P ≤ 0.05 compared to mock.

CELF-mediated alternative splicing has been implicated in regulating contractile function in the heart [31,32,47]. A CELF1 CLIP tag was identified that maps to the first annotated intron of the chick *MYH7B* gene (Fig 3A). *MYH7B* encodes a myosin heavy chain protein expressed in the embryonic heart, which is incorporated into the thick filaments of the myofibril [48]. To determine whether CELF1 binding could regulate the inclusion of the adjacent downstream exon, semi-quantitative RT-PCR was performed using primers in the flanking exons on total RNA from primary chicken embryonic cardiomyocytes following CELF1 knockdown. Alternative splicing of the annotated upstream exon could not be assessed by RT-PCR, as any sequences upstream of this exon in chick transcripts remain unknown. Surprisingly, although the downstream exon was constitutively included, a second, unexpected PCR product of slightly higher molecular weight was identified (Fig 3B). Gel isolation and sequencing of the PCR products and alignment against the chick *MYH7B* genomic sequence revealed the variable inclusion of a previously unannotated 84 nt exon located just 27 nt upstream of the CLIP tag (Fig 3A). Translation of the transcript sequences indicates that inclusion of this exon would lead to the insertion of an in-frame stop codon (Fig 3C). Transfection of primary embryonic cardiomyocytes with either of two siRNAs (si1 and si2) against CELF1 reduces its protein levels, with si2 providing a much more robust knockdown than si1, while a non-targeting control siRNA (siCont) has no effect on CELF1 levels [27]. The level of inclusion of this exon inversely correlates with CELF1 protein levels in chick primary embryonic cardiomyocytes (Fig 3B), suggesting CELF1 inhibits exon inclusion. This is in contrast to the RNA binding map generated for CELF1 in skeletal muscle, where negative regulation of exon inclusion by CELF1 is associated with binding in the upstream intron, and enhanced inclusion is associated with binding downstream [15].

The stop codon encoded by the novel alternative exon is close to the N-terminus of the protein, preceding the first known functional domain. Its inclusion, therefore, could lead to the production of a truncated, nonfunctional myosin peptide. Alternatively, inclusion of this exon could lead to destabilization of the transcript via the nonsense mediated decay (NMD) pathway, which recognizes transcripts containing premature termination codons and targets them for destruction [49]. It should be noted that if this is the case, the relative fraction of *MYH7B* transcripts including this exon may be higher than shown in Fig 3B, as unstable transcripts would be under-represented in measurements of steady state levels. Total *MYH7B* transcript levels are indeed lower in primary cardiomyocytes when exon inclusion is higher (Fig 3D), consistent with NMD.

### Knockdown of CELF1 in primary embryonic cardiomyocytes leads to extensive changes in cardiac gene expression

**Fig 4:**
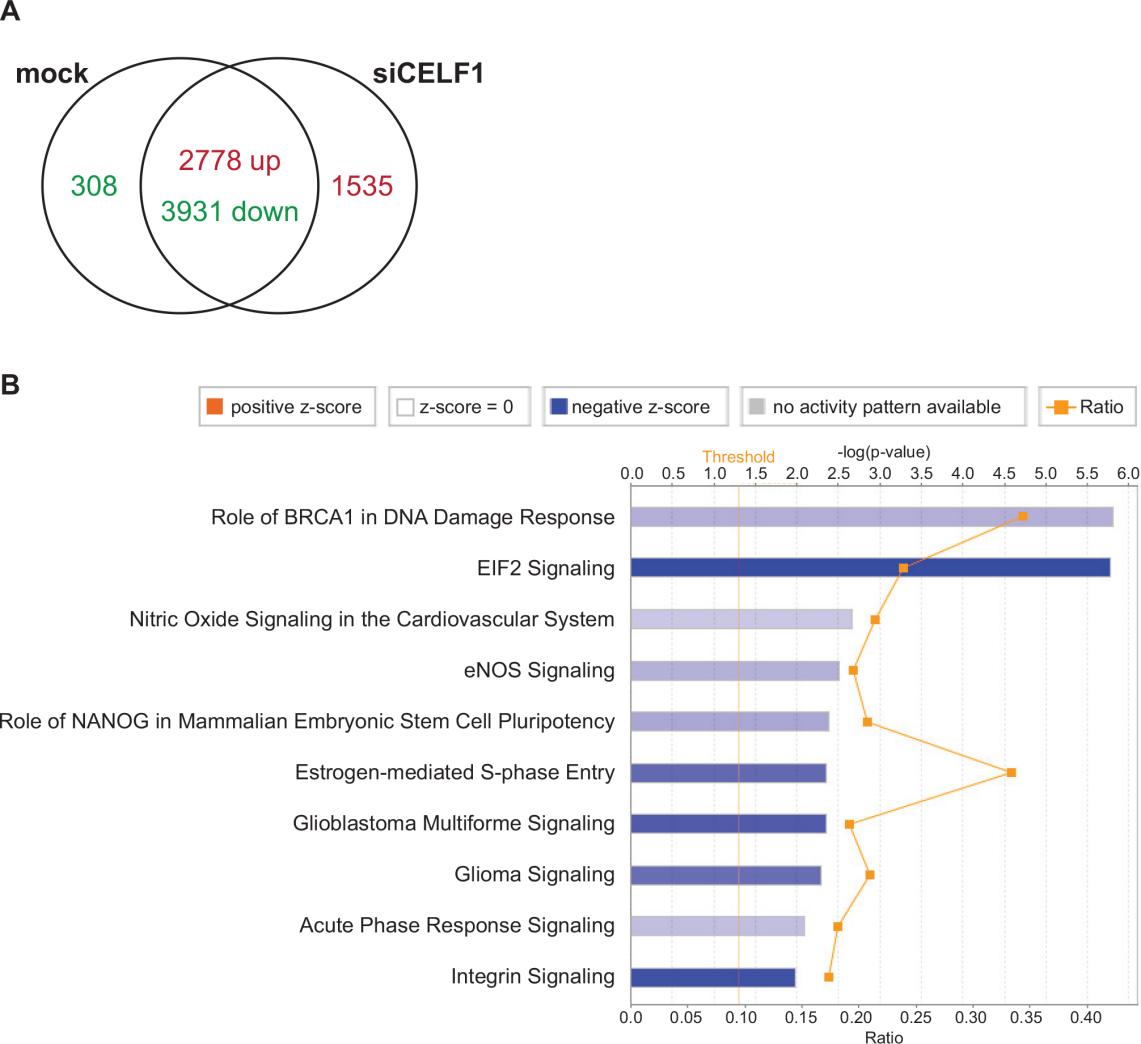
The transcriptome is substantially altered following CELF1 knockdown. (A) RNA-seq analysis of primary embryonic chicken cardiomyocytes ± siRNA directed against CELF1 (si2) identified a large number of transcripts that were down-regulated (green) or up-regulated (red) following CELF1 knockdown, including 308 transcripts that were expressed only in mock-transfected cells and 1535 transcripts that were expressed only in cells depleted of CELF1. (B) The ten top canonical pathways affected in primary embryonic chicken cardiomyocytes following CELF1 knockdown were identified using IPA software. The intensity of the color of each bar reflects the absolute value of the z-score for that pathway; a negative z-score indicates that a pathway is predicted to be inactivated. The ratio indicates the fraction of molecules in a pathway that are significantly different following CELF1 knockdown.

To characterize CELF1-dependent changes in gene expression at a more global level, we analyzed a recently published RNA-seq dataset from primary chicken embryonic cardiomyocytes with and without siRNA-mediated knockdown of CELF1; a detailed description of how this RNA-seq dataset was generated can be found in Blech-Hermoni and Ladd [27]. Using a threshold of ≥1.5-fold absolute change, we identified 4239 transcripts that were down-regulated and 4313 transcripts that were up-regulated following CELF1 knockdown (Fig 4A and S3 Table). Of these, 6709 were expressed in both mock- and siCELF1-transfected cells, whereas 308 were expressed only in control cells and 1535 were detected only in cells with reduced CELF1 levels. Of the shared transcripts, approximately one third of those that were down-regulated (1345 out of 3931) were reduced more than 2-fold, and almost two thirds of those that were up-regulated (1811 out of 2778) were more than 2-fold higher following CELF1 knockdown.

**Fig 5:**
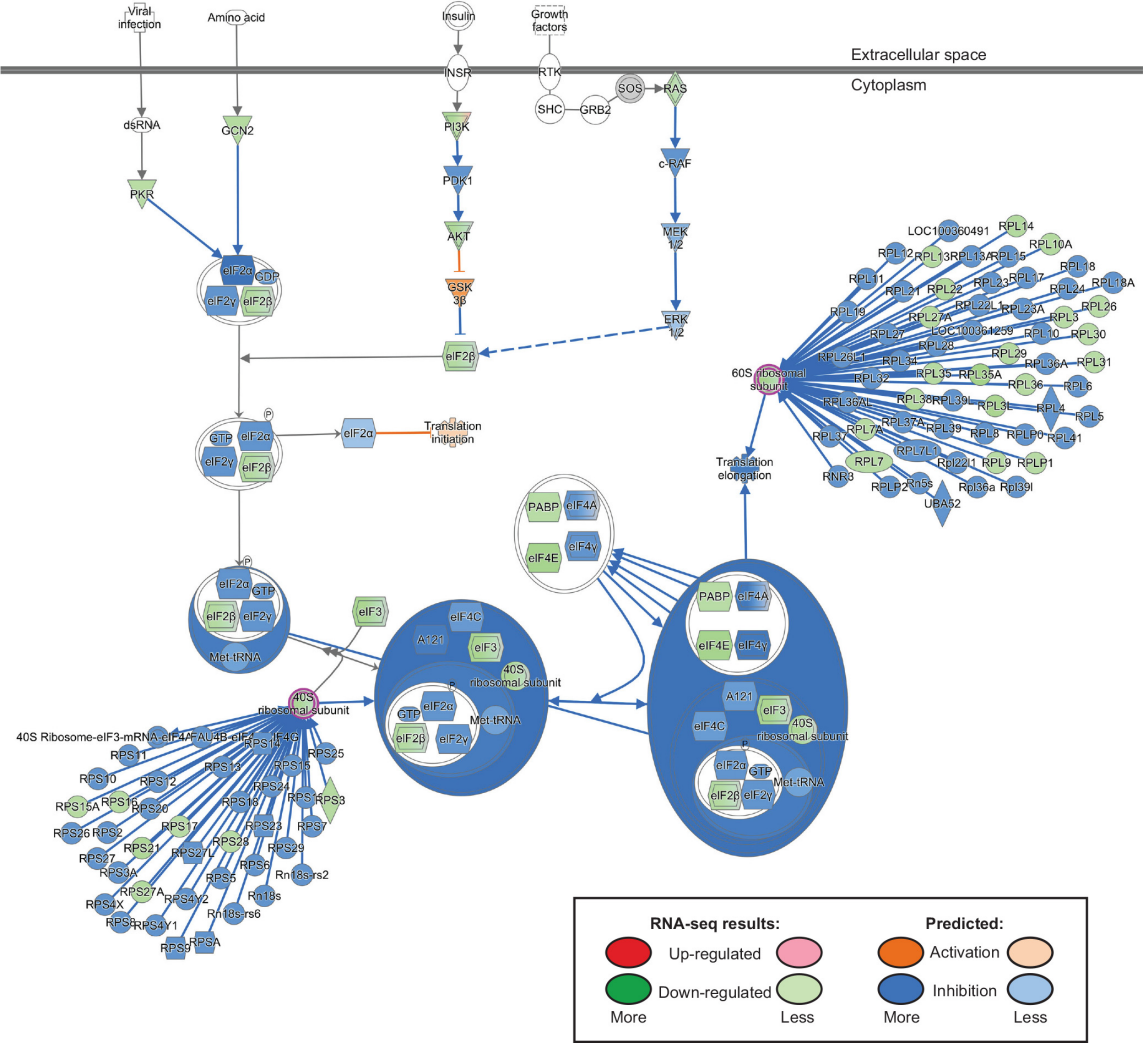
The EIF2 signaling pathway is strongly inhibited following knockdown of CELF1. The EIF2 signaling pathway was identified as one of the top canonical pathways affected by CELF1 knockdown using Ingenuity Pathway Analysis software. This pathway is shown with a molecule activity predictor (MAP) overlay from differential gene expression analysis of the RNA-seq dataset. Green indicates reduced expression in si2-transfected versus mock-transfected cardiomyocytes, whereas red/pink would indicate increased expression. Blue indicates predicted inhibition, whereas orange indicates predicted activation. The degree of saturation indicates the level of observed change or predicted effect. The 40S and 60S ribosome subunits (highlighted in magenta) have been expanded to show individual members of the complexes.

To better understand how embryonic cardiomyocytes are affected by loss of CELF1, pathway and network analyses were performed using Ingenuity Pathway Analysis software. The top biological functions affected by CELF1 knockdown include cell and tissue morphology, growth and proliferation, development, cell cycle, and cellular movement (Table 3). Network analysis also identified associations between loss of CELF1 and basic cellular processes including several networks related to the cell cycle (S4 Table). The ten top canonical pathways associated with CELF1 knockdown in our RNA-seq data are shown in Fig 4B. Of note, one of the pathways predicted to be strongly inhibited is EIF2 signaling. CELF1 has been shown to interact with the eIF2 initiation complex to promote translation of specific targets [50,51]. A closer examination of the canonical EIF2 signaling pathway reveals that CELF1 may have a general effect on translation elongation as well, as numerous components of both the 40S and 60S ribosomes are down-regulated following knockdown (Fig 5).

**Table 3:** Top functions identified by Ingenuity Pathway Analysis software as affected by CELF1 knockdown.

Top Bio Functions	Name	p-value	# Molecules
Molecular and Cellular Functions			
	Cell Morphology	3.30E-19–1.11E-04	580
	Cellular Growth and Proliferation	5.48E-19–1.53E-04	868
	Cellular Development	3.01E-16–1.53E-04	779
	Cell Cycle	5.36E-15–1.42E-04	362
	Cellular Movement	1.79E-14–1.35E-04	522
Physiological System Development and Function			
	Organismal Survival	2.35E-14–4.75E-05	558
	Cardiovascular System Development and Function	8.62E-14–1.50E-04	352
	Organismal Development	8.72E-14–1.53E-04	800
	Nervous System Development and Function	1.87E-13–1.46E-04	422
	Tissue Morphology	5.45E-13–1.27E-04	601

### Knockdown of CELF1 affects the expression of other regulatory proteins

**Fig 6:**
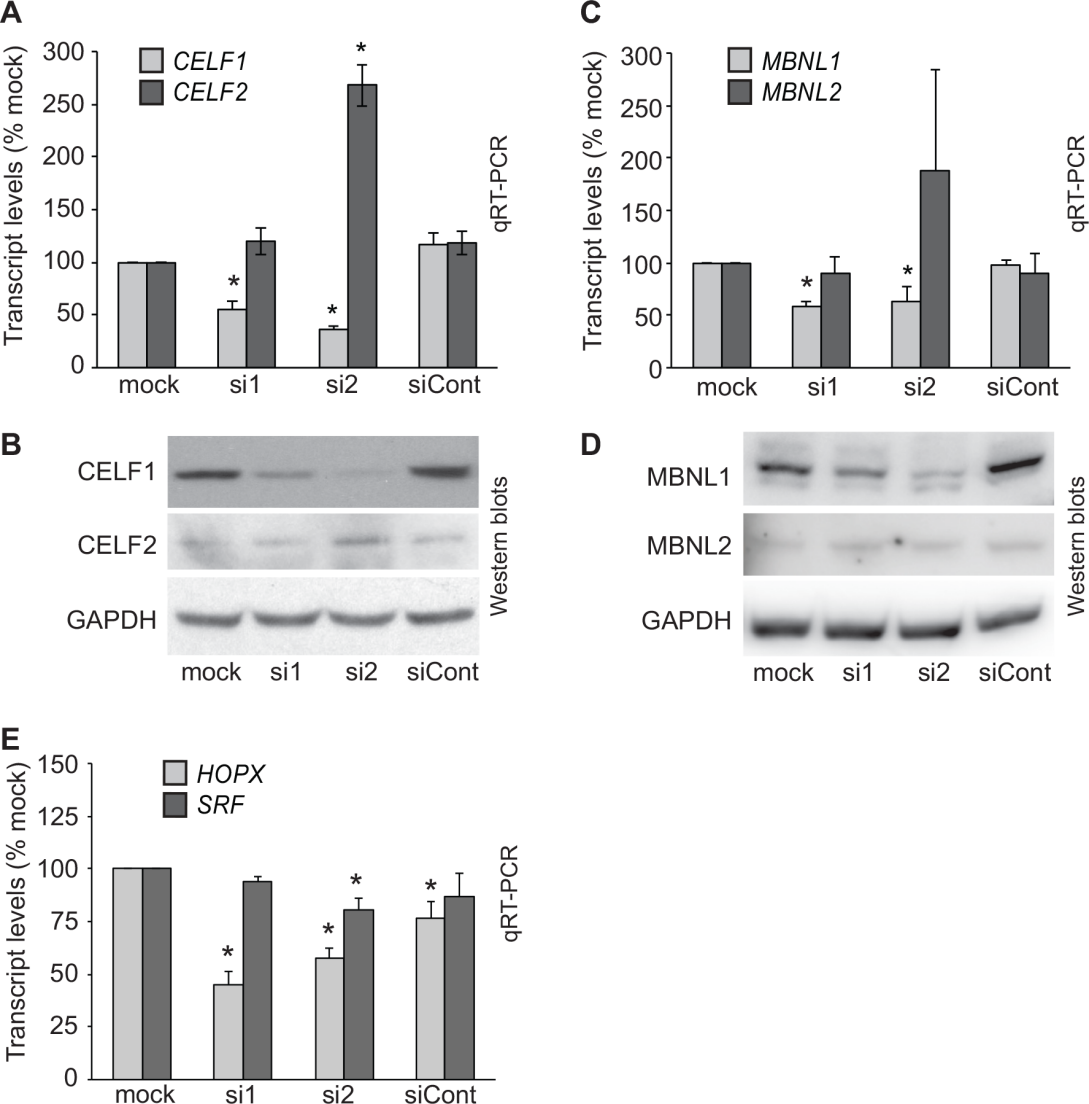
Knockdown of CELF1 affects the expression of other regulatory proteins. (A) Total *CELF1* and *CELF2* transcript levels were determined by qRT-PCR in primary embryonic cardiomyocytes transfected with or without siRNAs against CELF1 (si1 and si2) or a control siRNA (siCont). Data represent mean values from three independent transfections. An asterisk indicates P ≤ 0.05 compared to mock. (B) Western blots were performed on total protein samples collected from primary embryonic cardiomyocytes transfected in parallel to those used to harvest RNA. A non-specific 72 kDa band that does not vary between treatments was also observed on some CELF2 blots (data not shown). Representative blots from one of five independent transfections are shown. (C) Total *MBNL1* and *MBNL2* transcript levels were determined by qRT-PCR in primary embryonic cardiomyocytes ± si1, si2, or siCont. Data represent mean values from four independent transfections. An asterisk indicates P ≤ 0.05 compared to mock. It should be noted that the high standard error and apparent trend towards up-regulation in *MBNL2* transcript levels in si2-treated cells is the result of a single outlier. (D) Western blots were performed on total protein samples collected from primary embryonic cardiomyocytes transfected in parallel to those used to harvest RNA. Representative blots from one of four independent transfections are shown. (E) Total *HOPX* and *SRF* transcript levels were determined by qRT-PCR in primary embryonic cardiomyocytes ± si1, si2, or siCont. Data represent mean values from three independent transfections. An asterisk indicates P ≤ 0.05 compared to mock.

In a study using the C2C12 mouse skeletal muscle cell line, CELF1 was shown to bind to the 3’ UTRs and negatively regulate the half-lives of transcripts encoding other RNA binding proteins, including CELF2, a closely related paralog of CELF1 [15]. CELF2 is also expressed in the embryonic myocardium [3–5]. The RNA-seq data confirmed that while *CELF1* transcript levels were reduced greater than 2.5-fold in primary embryonic cardiomyocytes following CELF1 knockdown, *CELF2* transcript levels were elevated. We also evaluated CELF transcript levels by real-time RT-PCR and protein levels by western blot. Indeed, *CELF2* transcript levels were significantly elevated following transfection with the siRNA that yields the more robust knockdown of *CELF1* (si2), although not with the siRNA that yields more modest knockdown (si1; Fig 6A). By western blot, CELF2 protein levels were likewise consistently elevated following knockdown with si2 (Fig 6B). A slight increase in CELF2 protein levels were also seen following knockdown with si1 in some, but not all, transfection sets (data not shown). Thus, loss of CELF1 induces a proportionate increase in CELF2 in primary embryonic cardiomyocytes.

Like *Celf2*, CELF1 binding sites have been identified in the 3’ UTRs of *Mbnl1* and *Mbnl2*, and knockdown of CELF1 in C2C12 cells resulted in an increase in the half-lives of these transcripts [15]. MBNL1 and MBNL2 are members of the muscleblind-like (MBNL) family of RNA binding proteins, which regulate pre-mRNA alternative splicing, alternative polyadenylation site usage, mRNA stability and localization [15,52–54]. MBNL1 and MBNL2 are also expressed in embryonic heart muscle [3,30,55]. In our RNA-seq data, *MBNL1* levels were lower, not higher, following CELF1 knockdown, and *MBNL2* was not identified as differentially expressed. Consistent with our RNA-seq results, real-time RT-PCR confirmed that *MBNL1* transcripts were reduced in primary embryonic cardiomyocytes transfected with siRNAs against CELF1, while *MBNL2* transcript levels were not significantly affected (Fig 6C). Likewise, western blots showed a decrease in MBNL1 protein levels following CELF1 knockdown, but no change in MBNL2 (Fig 6D). Thus, in contrast to C2C12 cells where CELF1 promotes decay of *MBNL* transcripts, CELF1 is required to maintain levels of MBNL1 and has no effect on MBNL2 in primary embryonic cardiomyocytes.

In addition to regulating RNA binding proteins, CELF1 also regulates cardiac transcription factors. We previously reported that in MHC-CELFΔ mice, which express a dominant negative CELF protein in heart muscle, levels of homeodomain only protein x (*HOPX*) and four and a half LIM domain-containing protein 2 (*FHL2*) were reduced [31]. HOPX and FHL2 bind to the transcription factor serum response factor (SRF) and inhibit its activity [56–58], and indeed loss of HOPX and FHL2 in MHC-CELFΔ hearts is accompanied by an up-regulation of SRF target genes despite similar levels of SRF [31,59]. To determine whether CELF1 similarly regulates this transcription program in chicken embryonic cardiomyocytes, we examined *HOPX*, *FHL2*, and *SRF* in the RNA-seq dataset. *HOPX* transcript levels were reduced 2.0-fold following CELF1 knockdown (S3 Table). This reduction was confirmed for both CELF1 siRNAs by real time RT-PCR, although *HOPX* levels were also slightly reduced by the non-targeting control siRNA (Fig 6E). Surprisingly, the *FHL2* gene is not annotated in the chicken, and transcripts were not identified in the RNA-seq dataset that mapped to the locus in the chicken genome identified as orthologous to mammalian *FHL2* genes. As expected, *SRF* was not identified as differentially expressed in the RNA-seq data, but showed a slight reduction (~20%) following robust knockdown of CELF1 by real time RT-PCR (Fig 6E). Thus, modulation of the SRF transcription program via CELF-mediated regulation of inhibitory proteins is partially conserved between mouse and chicken.

## Discussion

The identification of transcripts and pathways that are subject to CELF1 regulation in heart muscle is an important step towards understanding its role in cardiomyopathies. The involvement of CELF1 in cardiac pathogenesis has most extensively been studied in DM1. DM1 is a genetic disorder caused by the expansion of CUG repeats in the 3’ UTR of *DMPK* transcripts, which leads to the dysregulation of other cellular transcripts [60]. CELF1 is normally down-regulated in the heart during postnatal maturation [1,3], but is elevated in the hearts of DM1 patients [8], and is rapidly up-regulated in heart muscle following induction of expanded CUG repeat-containing RNA in a DM1 mouse model [9]. The up-regulation of CELF1 in the adult heart in these mice mimics normal levels of CELF1 in the fetal heart [1,3,30], and fetal alternative splicing patterns are reiterated in adult DM1 tissues [7–9]. CELF1 is also up-regulated in a mouse model of diabetic cardiomyopathy, and has been proposed to similarly reactivate fetal alternative splicing in the diabetic heart [12]. Although the effects on cytoplasmic targets of CELF1 have not been investigated in these cardiomyopathies, dysregulation of CELF1-dependent translation is known to contribute to skeletal muscle pathology in DM1 [20]. In this study, we used a two-pronged approach to identify CELF1 targets in the developing heart. First, we performed CLIP for CELF1 on mid-gestation embryonic chicken hearts. Second, we analyzed RNA-seq data from chicken primary embryonic cardiomyocytes ± CELF1 knockdown. CLIP provides information about which transcripts are bound by CELF1, whereas RNA-seq provides information about the effects of CELF1 on global transcript levels. In the future, we hope the targets of CELF1 regulation identified here may prove useful for the design of new therapies to prevent or ameliorate cardiomyopathy in patients with DM1 or diabetes.

CELF1 CLIP had previously been reported from postnatal mouse brain [24], and CLIP-seq from mouse C2C12 myoblasts [15]. Consistent with these reports and *in vitro* binding studies [39–42], we found that CELF1 binds to sequences enriched in UG dinucleotide motifs. Although the majority of CELF1 CLIP tags fall within introns, which would be consistent with CELF1’s localization in the nucleus in myocardial cells [4] and its role as a regulator of pre-mRNA alternative splicing in the heart [1–3], binding alone does not necessarily denote regulation. It was recently reported that only about 12% of exons predicted to undergo MBNL2-dependent splicing regulation based on MBNL2 CLIP tag clusters exhibited altered splicing in microarrays using exon junction probes or RNA-seq data from *Mbnl2*-null mice [61]. The predominance of CELF1 binding in large introns far from known splice sites may suggest that a large percentage of intronic CELF1 binding is in fact nonfunctional. Given their propensity to bind to short, degenerate motifs, it is not surprising that binding of CELF1 and other RNA binding proteins can be found within very large introns, which would be likely to harbor such motifs merely by chance. The UV cross-linking used in the CLIP method has the potential to capture transient, low affinity interactions as well as stable, high affinity interactions. That is not to say that such binding would necessarily be without consequences within the cell. A large number of nonfunctional binding sites could act as a molecular sponge, “soaking up” and limiting the amount of an RNA binding protein available to interact with its targets. Both coding and non-coding RNAs have been proposed to act as molecular sponges for microRNAs, reducing the available pool that can bind and regulate their target mRNAs [62]. Alternatively, binding within these large introns could function to repress the use of pseudo splice sites, regulate as yet unannotated exons (such as the novel exon we identified in *MYH7B*), or act on true splice sites at a distance. Although most known splicing regulation occurs via binding to proximal elements [63], recent studies have shown that distal intronic elements can dictate splice site choice in some cases [64,65].

We identified a novel CELF1-regulated exon in the first intron of the chicken *MYH7B* gene that had a proximal CELF1 CLIP tag located 27 nt downstream. Inclusion of this exon is predicted to target *MYH7B* transcripts for NMD, and indeed a decline in *MYH7B* levels is observed when inclusion of this exon is stimulated by CELF1 knockdown in chicken primary embryonic cardiomyocytes. MYH7B has been shown to incorporate into the sarcomere, but how MYH7B is distinct from other myosin heavy chain proteins in the contractile apparatus is unknown. *MYH7B* expression is up-regulated during induced cardiac hypertrophy in mice [48], and a loss-of-function mutation in the *MYH7B* gene has been proposed to underlie congenital myopathy with left ventricular non-compact cardiomyopathy when combined with a mutation in the *integrin alpha 7* (*ITGA7*) gene in human patients [66]. These data suggest that expressing the right level of MYH7B in heart muscle is important for healthy contractile function. Using the conservation track of the UCSC genome browser, the region of the chicken *MYH7B* gene containing the novel exon and CLIP tag shows poor conservation in mammalian species. By RT-PCR we found no evidence of an alternative exon in the same relative position in the mouse *Myh7b* gene (data not shown). Differential splicing of *MYH7B* transcripts could reflect different regulatory mechanisms employed in chicken versus mouse. Some differences in *MYH7B* expression patterns have been reported between species [48]. Another group reported that skipping of a downstream exon, exon 7, leads to the introduction of a premature termination codon and down-regulation of *Myh7b* transcripts in mouse cells [67]. It is therefore also possible that the different species employ a similar mechanism yet use different exons. An advantage of splicing-based NMD as a mechanism for regulating *MYH7B* levels is that it enables the cell to separate steady state levels of MYH7B protein from transcription of the *MYH7B* gene, which also hosts a microRNA, miR-499, within one of its introns [67].

By comparing the transcriptomes of CELF1-depleted and control cells in a published RNA-seq dataset [27], we found more than 8,000 transcripts whose levels differed by at least 1.5-fold, and over 3,000 that differed by at least 2-fold. These should include both transcripts directly bound and regulated by CELF1, and those that lie downstream of direct CELF1 targets. Although we did not identify CELF1 CLIP tags in the majority of the differentially expressed transcripts, it should be noted that the number of CLIP tags sequenced in this study is likely far from saturating. Network analysis found a strong association between CELF1 and cell cycle, consistent with previous studies linking CELF1 to cell cycle regulation and proliferation during skeletal muscle differentiation, T cell activation, oocyte maturation, and cancer [18,68–74]. Pathway analysis also revealed that many transcripts encoding ribosomal proteins within both the 40S and 60S subunits are down-regulated following CELF1 knockdown. Thus, in addition to regulating transcript levels and variants, CELF1 may play a broader role in regulating translation than previously appreciated.

In the heart, CELF1 and CELF2 protein levels rise, peak, and then fall together during embryonic and postnatal development, while their transcript levels remain constant [1,3,4]. Our data suggest, however, that there is a feedback mechanism that stimulates *CELF2* transcript (and subsequent protein) levels when CELF1 activity is low. Other splicing factors exhibit similar behavior. For example, MBNL2 levels are elevated in some tissues in *Mbnl1*-knockout mice and vice versa [75,76]. Although CELF1 and CELF2 bind to similar U/G-rich sequences [40,77], not all targets are shared between them [78]. The extent to which the up-regulation of CELF2 alleviates–or causes–changes in gene expression following CELF1 knockdown remains to be determined. Loss of CELF1 also led to altered levels of other regulatory factors, such as decreases in both MBNL1 and HOPX. Loss of MBNL1 function has been implicated in DM1 pathogenesis [60], and many cardiac transcripts display altered splicing patterns in *Mbnl1*-knockout mice [76,79,80]. HOPX inhibits the activity of SRF, and loss of HOPX is sufficient to induce an up-regulation of some SRF target genes in mice [58]. SRF regulates the transcription of a large number of cardiac genes involved in contractile function, and plays roles in normal development, pathogenesis, and aging in the heart [81–83]. *HOPX* is down-regulated in human heart failure [84], and ablation of HOPX causes cardiac hypertrophy in mice [58]. Thus, CELF1 likely regulates cardiac gene expression both directly, through binding to its target transcripts, and indirectly, through the regulation of other RNA and DNA binding proteins.

## Supporting Information

S1 Fig Sequence enrichment in Nova CLIP tags differs from CELF1 CLIP tags. The analysis of multimer frequency that was performed on our CELF1 CLIP tags was performed on a published set of Nova CLIP tags from adult mouse hindbrain. CLIP tag data set taken from Ule, et al. 2003. (A) Incidence of dinucleotides within Nova CLIP tags. The dotted green line indicates the incidence expected if all dinucleotides were equally represented. Dinucleotides found in the known Nova binding element, YCAY, are in red. (B) Incidence of hexanucleotides within Nova CLIP tags from adult mouse hindbrain. The ten most-frequent hexanucleotides are indicated in red. The dotted green line indicates the incidence expected if all hexanucleotides were equally represented. The sequences of the top hexanucleotides are shown, with YCAY motifs in red.
(PDF)

S1 Table CELF1 CLIP tags obtained from embryonic day 8 chicken heart (XLS)

S2 Table The subset of intronic CELF1 CLIP tags that fall within annotated genes (XLS)

S3 Table Transcripts exhibiting significant differences in expression of ≥ 1.5-fold between mock- and siCELF1-transfected primary embryonic cardiomyocytes (XLSX)

S4 Table Networks identified as affected in siCELF1 (si2)- compared to mock-transfected primary embryonic cardiomyocytes by Ingenuity Pathway Analysis software (XLS)

S1 File Extended Materials and Methods. Detailed methods are provided for performing CELF1 cross-linking immunoprecipitation (CLIP) from embryonic chicken hearts.
(DOC)

S2 File Script to extract CLIP tag sequences from clone sequences. (PL)

S3 File Script to count the occurrence of each nucleotide combination (1- to 6-mer) in CLIP tags. (PL)

S4 File Script to compare the genomic coordinates of CLIP tags to introns. (PL)
