# Supplementary figures and images for: Identification of Targets of CUG-BP, Elav-Like Family Member 1 (CELF1) Regulation in Embryonic Heart Muscle
Source: PLoS One. 2016 Feb 11;11(2):e0149061. doi: 10.1371/journal.pone.0149061 (PMC4750973; doi:10.1371/journal.pone.0149061)

Figure S1

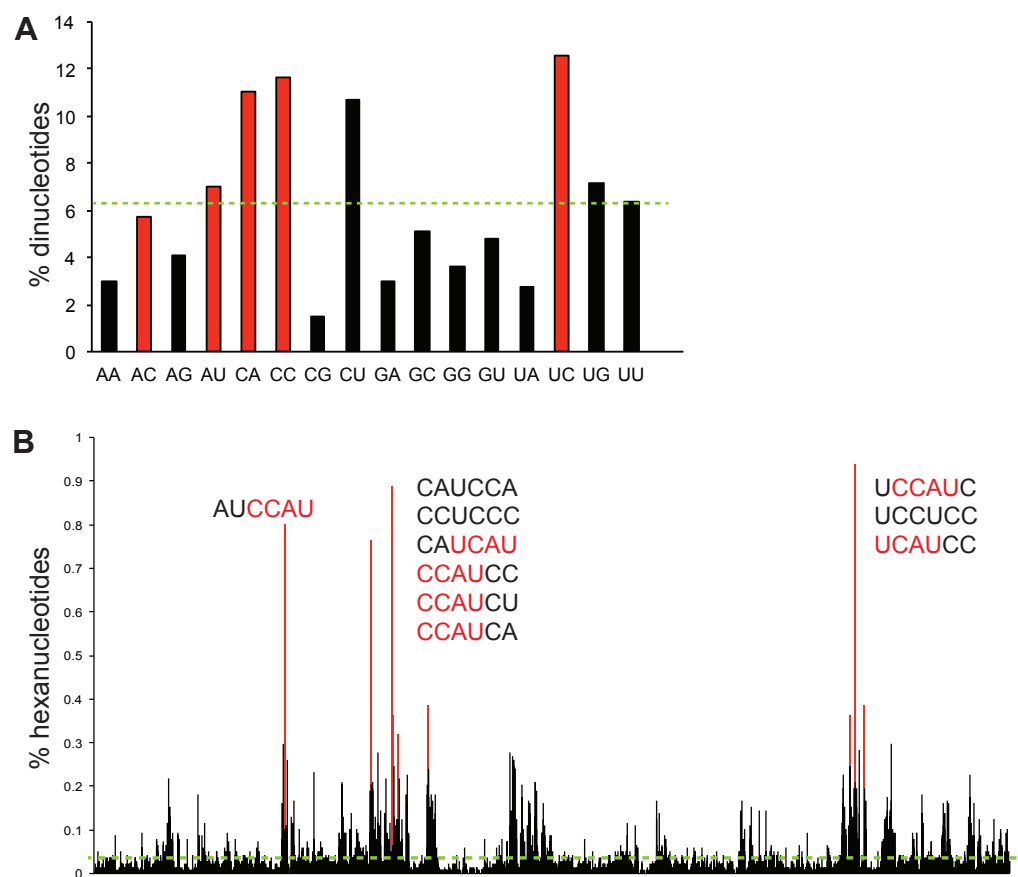

Supplement: S1 Fig — The analysis of multimer frequency that was performed on our CELF1 CLIP tags was performed on a published set of Nova CLIP tags from adult mouse hindbrain. CLIP tag data set taken from Ule, et al. 2003. (A) Incidence of dinucleotides within Nova CLIP tags. The dotted green line indicates the incidence expected if all dinucleotides were equally represented. Dinucleotides found in the known Nova binding element, YCAY, are in red. (B) Incidence of hexanucleotides within Nova CLIP tags from adult mouse hindbrain. The ten most-frequent hexanucleotides are indicated in red. The dotted green line indicates the incidence expected if all hexanucleotides were equally represented. The sequences of the top hexanucleotides are shown, with YCAY motifs in red. (PDF) [file pone.0149061.s001.pdf]
