# Supplementary material for: Identification of Targets of CUG-BP, Elav-Like Family Member 1 (CELF1) Regulation in Embryonic Heart Muscle
Source: PLoS One. 2016 Feb 11;11(2):e0149061. doi: 10.1371/journal.pone.0149061 (PMC4750973; doi:10.1371/journal.pone.0149061)
Supplement: S1 File — Detailed methods are provided for performing CELF1 cross-linking immunoprecipitation (CLIP) from embryonic chicken hearts. (DOC) [file pone.0149061.s006.doc]

**Extended Materials and Methods**

**Cross-linking immunoprecipitation (CLIP)**

CLIP was performed following the general method of Ule and colleagues , with appropriate optimization for our protein (CELF1) and tissue (embryonic heart) at each step. Approximately 180-200 embryonic day 8 chicken hearts were collected in 4 ml ice-cold PBS, gently triturated, and transferred on a petri dish; the depth of the suspension was approximately 1 mm. The suspension was irradiated three times for 400 mJ/cm^2^ in a Stratalinker (Stratagene model 1800) on ice, mixing between each irradiation. After irradiation, the suspension was immediately pelleted by centrifugation at 3000 rpm for 5 min at 4°C. Cross-linked pellets were frozen at -80°C until ready to use. Cross-linked lysate was resuspended in Buffer A (1X PBS, 0.1% SDS, 0.5% deoxycholate, 0.5% NP-40). Superase.In (Ambion) was added to each tube to block RNA over-digestion. A “high RNase” sample treated with 1:100 RNase A (USB) was used as a marker. The samples were sonicated and each tube was treated with 30 µl RQ1DNase (Promega) for 5 min at 37 ˚C. These were ultracentrifuged for 20 min at 60,000 rpm at 4 ˚C, and supernatants were used for IP.

Dynabeads (Dynal) were equilibrated in Buffer A. Supernatant was incubated with anti-CELF1 antibody (3B1, Santa Cruz) for 45 min at room temperature with rotation, then washed three times with Buffer A and loaded onto the equilibrated beads. Following a one hour incubation at 4˚C, the beads were washed with Buffer A followed by a high salt buffer B (5X PBS, 0.1% SDS, 0.5% deoxycholate and 0.5% NP-40), and finally twice with 1X PNK buffer (50 mM Tris-Cl, pH7.4, 10mM MgCl_2_, 0.5% NP-40). An on-bead CIP (Roche) treatment was performed. The beads were incubated for 10 min at 37 ˚C at 1000 rpm, then washed with 1X PNK-EGTA buffer (50 mM Tris-Cl, pH7.4, 20mM EGTA, 0.5% NP-40) followed by 1X PNK buffer.

The 3’ linker was ligated on bead with T4 RNA ligase overnight at 16˚C. The following day the beads were washed 9-10 times with 1X PNK buffer, then treated with T4 PNK enzyme (NEB). Beads were washed three times with 1X PNK buffer. 40 µl of 1X PNK buffer and 40 µl Nu-PAGE LDS sample buffer (Life technologies) were added to the beads and boiled for 5 min. 1 µl β-mercaptoethanol was added to every 40 µl of the sample and boiled again. 40 µl supernatant from each tube was loaded onto a Novex NuPAGE 10% Bis-tris SDS-PAGE gel and transferred onto a nitrocellulose membrane. Smeared bands in the range of 70-120 kDa were cut out into a single eppendorf. This was digested with proteinase-K in PK buffer (100 mM Tris-Cl, pH 7.5, 50 mM NaCl, 10 mM EDTA) for 20 min at 37˚C at 1000 rpm. This incubation was repeated after addition of 200 µl of PK buffer in 7 M urea. RNA was extracted with phenol:chloroform followed by a 1:1 ethanol:isopropanol precipitation.

The 5’ linker was ligated to the resuspended RNA with T4 RNA ligase for 4 hours at 16˚C. DNA was digested with RQ1 DNAse, and RNA was re-isolated by phenol:chloroform extraction and ethanol:isopropanol precipitation. Reverse transcription was performed with P3 primer using Superscript III (Invitrogen). PCR was performed using P3/P5 primers and Accuprime Pfx Supermix with the following program: 95 ˚C for 5 min, followed by 35 cycles of 95˚C for 20 sec/61˚C for 30 sec/68˚C for 20 sec, and a final extension of 68˚C for 5 min. Samples were run on a 10% denaturing polyacrylamide gel, and DNA fragments in the range from 70-110 bp were extracted using a QIAEX II kit (Qiagen). The isolated PCR products were amplified two more times using P3/P5 primers and Accuprime Pfx supermix. The products were desalted with GE G25 microspin columns (Amersham) and subcloned into a TOPO vector (Invitrogen). Although 384 clones were sequenced, a total of 736 sequences representing 564 unique tags were obtained due to concatamerization of the tags. CLIP tags were identified by alignment to the chick (galGal3) genome by BLAT search using the University of California, Santa Cruz (UCSC) genome browser and/or BLAST search of the NCBI database .

**References**

1. Ule J, Jensen K, Mele A, Darnell RB: **CLIP: a method for identifying protein-RNA interaction sites in living cells**. *Methods* 2005, **37**(4):376-386.

2. Kent WJ: **BLAT--the BLAST-like alignment tool**. *Genome Res* 2002, **12**(4):656-664.

3. Altschul SF, Madden TL, Schaffer AA, Zhang J, Zhang Z, Miller W, Lipman DJ: **Gapped BLAST and PSI-BLAST: a new generation of protein database search programs**. *Nuc Acids Res* 1997, **25**(17):3389-3402.
